# A Bayesian Approach to Identifying New Risk Factors for Dementia: A Nationwide Population-Based Study

**DOI:** 10.1097/MD.0000000000003658

**Published:** 2016-05-27

**Authors:** Yen-Hsia Wen, Shihn-Sheng Wu, Chun-Hung Richard Lin, Jui-Hsiu Tsai, Pinchen Yang, Yang-Pei Chang, Kuan-Hua Tseng

**Affiliations:** From the School of Pharmacy, College of Pharmacy, Kaohsiung Medical University (Y-HW, S-SW); Department of Psychiatry (J-HT) and Department of Neurology (Y-PC), Kaohsiung Municipal Ta-Tung Hospital, Kaohsiung Medical University; Department of Psychiatry, Kaohsiung Medical University Hospital, Kaohsiung Medical University (PY); and Department of Computer Science and Engineering, National Sun Yat-sen University (C-HRL, K-HT), Kaohsiung, Taiwan.

## Abstract

Dementia is one of the most disabling and burdensome health conditions worldwide. In this study, we identified new potential risk factors for dementia from nationwide longitudinal population-based data by using Bayesian statistics.

We first tested the consistency of the results obtained using Bayesian statistics with those obtained using classical frequentist probability for 4 recognized risk factors for dementia, namely severe head injury, depression, diabetes mellitus, and vascular diseases. Then, we used Bayesian statistics to verify 2 new potential risk factors for dementia, namely hearing loss and senile cataract, determined from the Taiwan's National Health Insurance Research Database.

We included a total of 6546 (6.0%) patients diagnosed with dementia. We observed older age, female sex, and lower income as independent risk factors for dementia. Moreover, we verified the 4 recognized risk factors for dementia in the older Taiwanese population; their odds ratios (ORs) ranged from 3.469 to 1.207. Furthermore, we observed that hearing loss (OR = 1.577) and senile cataract (OR = 1.549) were associated with an increased risk of dementia.

We found that the results obtained using Bayesian statistics for assessing risk factors for dementia, such as head injury, depression, DM, and vascular diseases, were consistent with those obtained using classical frequentist probability. Moreover, hearing loss and senile cataract were found to be potential risk factors for dementia in the older Taiwanese population. Bayesian statistics could help clinicians explore other potential risk factors for dementia and for developing appropriate treatment strategies for these patients.

## Abstract 2

Supplemental Digital Content is available in the text

## INTRODUCTION

Dementia is one of the most disabling and burdensome health conditions worldwide. Globally, 35.6 million people were estimated to be affected with dementia in 2012, and this number is expected to increase to 60 million by 2030 and to 114 million by 2050.^1,2^ In addition to its rapidly increasing incidence, dementia is the major cause of disability in the elderly population and imposes a huge economic burden worldwide.^3,4^ Several risk factors for dementia, such as advanced age, head injury, depression, diabetes mellitus (DM), and vascular diseases, have been recognized.^5–7^ However, other potential risk factors that might be useful for clinicians for developing appropriate treatment strategies in patients at the early stages of dementia and preventing the worsening of the condition remain either controversial or ignored.

Bayesian statistics was introduced in medical research in 1982; however, the US Food and Drug Administration approved and issued a draft guideline for its application in clinical research only in 2010.^8^ Bayesian statistics, which is learning from evidence as it accumulates, is currently applied in all major areas of medical statistics, including clinical trials, epidemiology, meta-analyses and evidence synthesis, spatial modeling, longitudinal modeling, survival modeling, molecular genetics, and decision making for new technologies.^9^ This approach, in short, is a way to combine the past (prior) with present (current study) to make decisions about the future (posterior conclusions).^8^ To the best of our knowledge, few studies have focused on using Bayesian statistics to identify other potential risk factors for dementia.

Chinese characters are a type of hieroglyphics. The Chinese character for intelligence denotes good hearing, keen eyesight, and a strong heart or brain. Dementia is defined as a progressive global intellectual impairment occurring in clear consciousness.^10^ Heart and brain diseases, such as acute myocardial infarction and stroke, are generally associated with increased risks of dementia. We hypothesized that older people with decreasing intellect (or dementia) are associated with poor hearing (hearing loss) and poor eyesight (senile cataract).

In this study, we identified new potential risk factors for dementia from nationwide longitudinal population-based data by using Bayesian statistics. We first tested the consistency of the results obtained using Bayesian statistics with those of classical frequentist probability with the 4 recognized risk factors for dementia, namely severe head injury, depression, DM, and vascular diseases. Then, we used Bayesian statistics to verify 2 new potential risk factors for dementia, namely hearing loss and senile cataract, determined from the Taiwan's National Health Insurance Research Database (NHIRD).

## MATERIALS AND METHODS

### Data Sources and Ethical Consideration

Since its implementation in 1995, the Taiwan's National Health Insurance (NHI) program has been providing comprehensive, unified, and universal healthcare services to approximately 99% of the Taiwanese population.^11^ We used one of the subsets of the Taiwan's NHIRD, developed by the NHI program using 1995 to 2010 data and consisting of 1 million patients (approximately 5% of the total Taiwanese population) randomly selected in 2010. The NHIRD contains data on patients’ demographics, diagnoses, medication types, prescription dates, and dosages and durations of drug supply.

All data used for the present study are available after others apply to the Center for Biomedical Resources of National Health Research Institutes in Taiwan and should be submitted to the executive committee (for more information please you refer to http://nhird.nhri.org.tw/en/). Furthermore, this study protocol was also approved by an institutional review board (KMUHIRB-SV (II)-20150007), and informed consent was waived because of the use of previously stored deidentified medical information from the NHIRD.

### Study Design and Sampling

The study group consisted of patients with dementia diagnosed on the basis of the International Classification of Disease, Ninth Revision, Clinical Modification (ICD-9-CM) diagnostic criteria (ICD-9-CM codes 290, 294.1–294.2, 331.0, A210, A213, and A222) between March 1995 and December 2010. To enhance diagnostic validity, we only selected patients who had inpatient diagnosis files with primary or secondary diagnosis of dementia or outpatient diagnosis files with at least three consistent diagnosis of dementia.^12^ We assigned their first visit for the diagnosis of dementia as their index date.

### Potential Risk Factors Associated in Patients With Dementia

We assessed patients with dementia before or on their index date to ascertain their histories of severe head injury (ICD-9-CM codes 431, 430, 800–804, 850–852, 853.1–853.2, 854.0–854.1, A290, A291, and A470), depression (ICD-9-CM codes 296.2–296.3, 296.82, 293.83, 300.4, and 311), DM (ICD-9-CM codes 249–250, 648.01, 648.02, 588.1, 357.2, and A181), vascular diseases (ICD-9-CM codes 325, 430–459, 410, 411.0, 411.1, 411.81, 411.89, 412, 413.1, 413.9, 414.00–414.07, 414.10–414.12, 414.19, 414.2–414.4, 414.8, 414.9, A279, A291-A294, and A299), senile cataract (ICD-9-CM codes 366 and A231), and hearing loss (ICD-9-CM codes 389 and A241) (see Table e-1 in the Appendix;).^13–16^ In addition, we examined the inpatient and outpatient diagnosis files of patients without dementia between 1995 and 2010 to ascertain their histories of severe head injury, depression, DM, vascular diseases, senile cataract, and hearing loss. Sociodemographic data of enrolled patients was also recorded, including age, gender, geographic region, urban level, and monthly income (see Table 1).

**TABLE 1:**
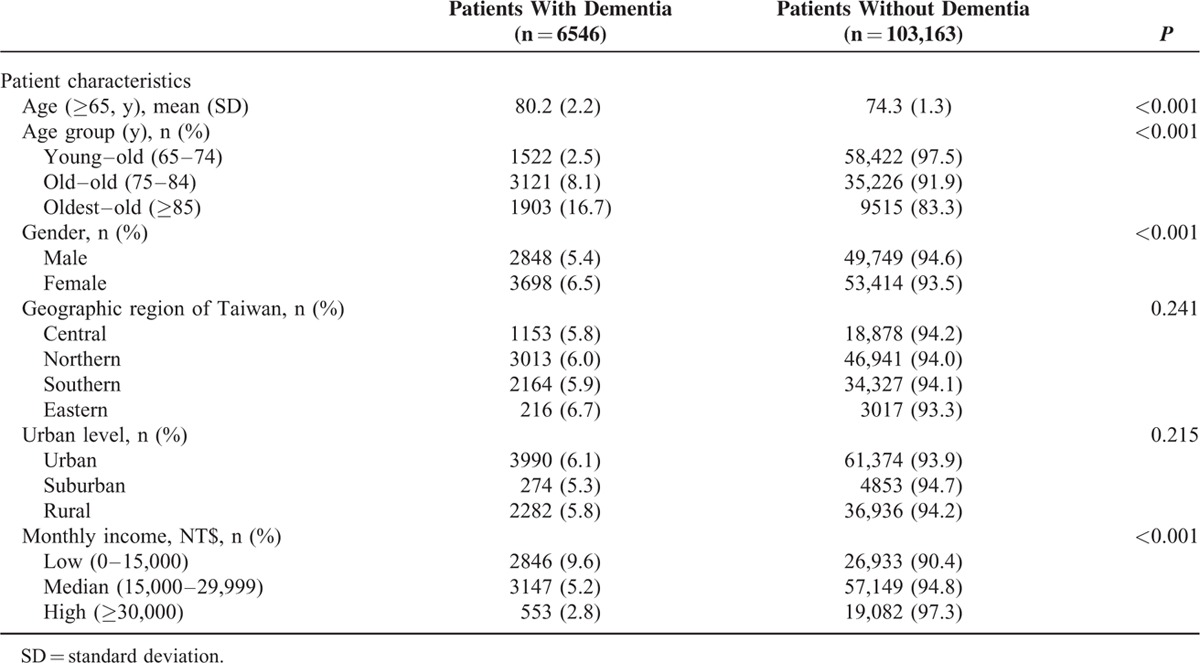
Sociodemographic Characteristics of the Enrolled Elders, Stratified by Presence or Absence of Dementia From 1995 to 2010 (N = 109,709)

### Statistical Analysis

We derived the odds ratio (OR) by using the Bayesian approach.^17^ We attempt to learn about the unknown distribution from given data, to make some inferences about the certain properties of the distribution, and to determine the relative likelihood that each possible distribution is actually the correct one. Suppose that proportion θ of patients with dementia in a population in the presence of a risk factor is unknown, and let the prior distribution assigned to θ be a uniform distribution at the interval (0, 1); that is, the prior p.d.f. ζ(θ) = 1 for 0 < θ < 1. This “informationless” prior assignment is for the sake of objective purpose and reducing computing cost. Suppose there is a given random sample of n persons who all have a risk factor, and for i = 1, 2, 3, n, let X_i_ = 1 if the *i*th person has dementia and let X_i_ = 0 otherwise. Then, X_1_, X_2,_ X_3_, X_n_ form n Bernoulli trials with parameter θ. We can determine the posterior p.d.f. of θ. The p.f. of each observation X_i_ is
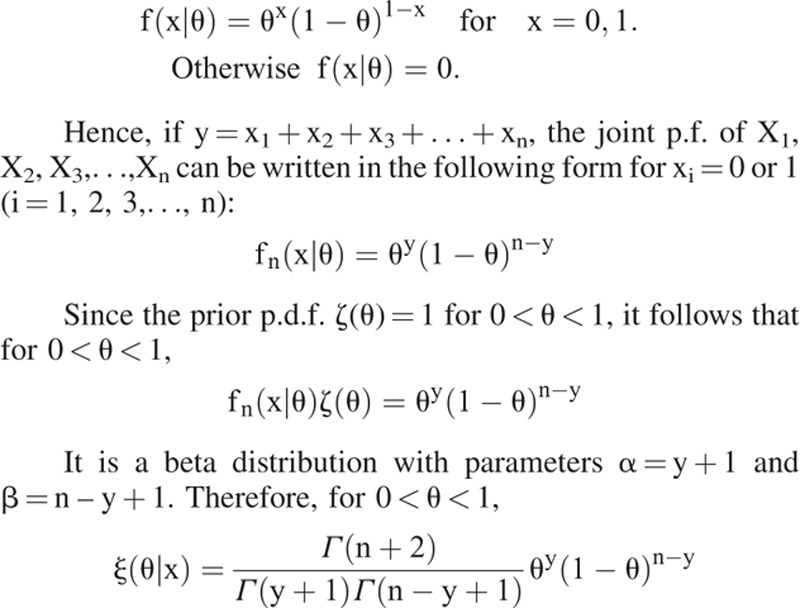


That is, the above derived result illustrates that the posterior distribution of the combination of uniform prior distribution and the likelihood function of Bernoulli trials is Beta distribution. The mean of θ given the observation x is E_n_

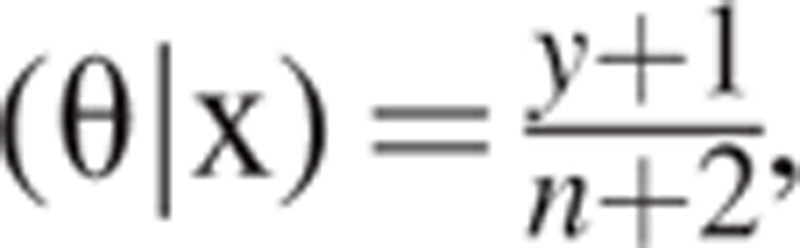
, which is also the estimate of θ based on the squared error loss function that is to be derived later. Let θ^∗^ be the estimate of θ; then, the OR is equal to 
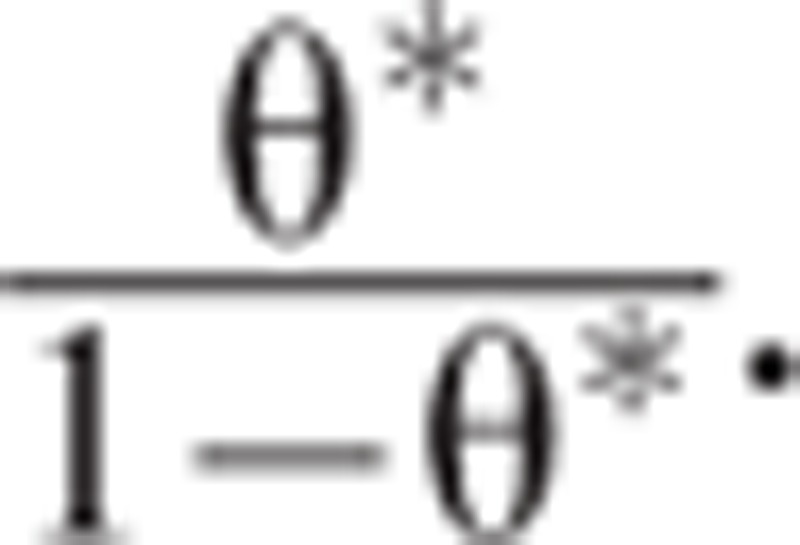
. Similarly, we can get the OR of cases in a population in the absence of a risk factor, following which the OR can be obtained.

After obtaining the posterior probability distribution of θ using the Bayes’ theorem, we can identify the Bayes estimator, δ(X_1_, X_2_, X_3_,…, X_n_), a real-valued function of random variables X_1_, X_2_, X_3_,…, X_n_ that specifies the estimated value of θ for each possible set of values of observed data X_1_, X_2_, X_3_,…, X_n_. An estimate, say a, is a specific value of δ(x_1_, x_2_, x_3_,…, x_n_) of the estimator determined using specific observed values x_1_, x_2_, x_3_,…, x_n_. By using the squared error loss function, which is the most commonly used loss function in estimation problem, L(θ, a) = (θ – a)^2^. Bayes estimate δ^∗^(x) for any observed value of x will be the value of a for which the expectation E[(θ – a)^2^|x] is minimum. This expectation is minimum when a is chosen to be equal to the mean E(θ|x) of the posterior distribution; in other words, when the squared error loss function is used, the Bayes estimator is δ^∗^(X) = E(θ|X). Therefore, we can use the mean of posterior probability distribution as the estimate.

We used the aforementioned Bayesian inference—given the evidence of 1 million population, sampled from the NHIRD—to determine the posterior probabilities of θ. The posterior distribution is insensitive to variations in the prior distribution; this insensitivity rapidly increases with the sample size.^18^ This is the reason why we do not use a strong subjective prior and just choose an informationless prior. Furthermore, this can also reduce the computation cost. The probability calculus does more than explaining how states of belief (i.e., the proportion θ of dementia in a population in the presence of a risk factor) decompose into prior and empirical (i.e., the NHIRD) elements; it also measures the probability θ relative to the NHIRD. The prior distribution of θ is a uniform distribution of subjective prior probabilities over the range (0, 1). Thus, using the Bayes’ theorem and the NHIRD sample, we calculated the corresponding posterior probability to be a beta distribution as stated earlier. Thus, the posterior probability is concentrated in a narrow region even if the prior probability curve is spread out, illustrating the considerable initial uncertainty regarding the parameter value.

## RESULTS

### Demographic Characteristics of the Study Population

Of the 1,000,000 patients screened, we included 109,709 (11.0%) patients aged ≥65 years. A total of 6546 (6.0%) patients were diagnosed with dementia. Table 1 shows the distribution of sociodemographic characteristics of the study population. Patients with dementia were significantly older than those without dementia (80.2 years vs 74.3 years, *P* < 0.001). Moreover, the percentage of patients with dementia in the young–old (65–74 years), old–old (75–84 years), and oldest–old (≥85 years) groups was significantly increased by 2.5%, 8.1%, and 16.7%, respectively (*P* < 0.001). The percentage of women with dementia was significantly higher than that of men with dementia (n = 3698, 6.5% vs 2848, 5.4%; *P* < 0.001); furthermore, patients with high income had significantly lower incidence of dementia than in those with lower income (2.8%, 5.2%, and 9.6%; *P* < 0.001).

### Risk Factors for Dementia

Regarding the 4 recognized risk factors for dementia, ORs in the older population with a history of severe head injury, depression, DM, and vascular diseases were 1.701, 2.637, 1.207, and 3.469, respectively (Table 2); ORs for these risk factors were higher in men with dementia than in women with dementia except for DM. Furthermore, we tested ORs of other potential risk factors for dementia, including senile cataract and hearing loss. We observed that hearing loss (OR = 1.577) and senile cataract (OR = 1.492) were associated with an increased risk of dementia in the older population. In terms of sex-based differences, ORs of these risk factors were generally higher in men with dementia than in women with dementia (Table 2).

**TABLE 2:**
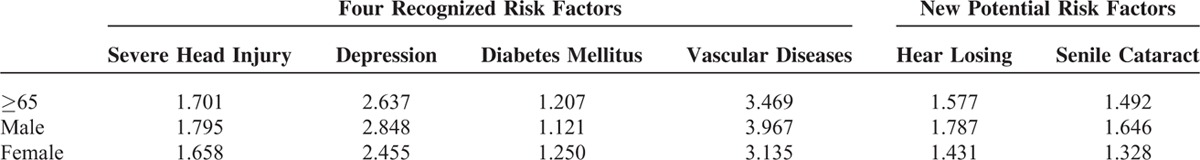
Risk Factors (Odds Ratios) for Dementia by Age and Gender

## DISCUSSION

To the best of our knowledge, this is the first nationwide population-based study identifying new potential risk factors for dementia using Bayesian statistics. Overall, older age, female sex, and lower income were identified as independent risk factors for dementia in this study. By using Bayesian statistics to assess the older population in Taiwan, we reidentified the 4 recognized risk factors for dementia, namely vascular diseases, depression, severe head injury, and DM; their ORs ranged were from 3.469 to 1.207. The results obtained are in agreement with our hypothesis that the 4 recognized risk factors for dementia using classical frequentist probability are consistent with those using Bayesian statistics. Furthermore, we identified that hearing loss (OR = 1.577) and senile cataract (OR = 1.492) are associated with an increased risk for dementia.

Taiwan has been an aging country since 1993, and the older population exceeded 10% of the total population in 2005.^5^ Our results showed that approximately 11% of the population was aged ≥65 years in 2010. The prevalence of dementia in the older population in Taiwan from 1995 to 1998 was 3.2%, which is lower than that in other developed countries.^5,19–22^ Thus, our data demonstrated that the prevalence of dementia in the older population from 1995 to 2010 was approximately 6%, similar to that in other developed countries.^20–22^ The risk of dementia for an individual can vary with area, population, and diagnostic criteria because various sociodemographic risk factors vary, expect for age and educational level. A review on Asian populations, including that of Taiwan, has showed that older age, female sex, and lower socioeconomic status were the risk factors for dementia in the East.^19^ These results are similar to those observed in our study.

Increasing evidence supports that increased risks of dementia are associated with its treatable medical comorbidities, including severe head injury,^5,23–25^ depression,^26,27^ DM,^24,28,29^ and vascular diseases.^5,30,31^ An analysis of 15 case–control studies from 1991 to 2001 concluded that a history of head injury is a significant risk factor for Alzheimer disease (AD, OR = 1.58), and OR for this risk factors is higher in men than in women.^23,24^ However, the OR of this study is lower than that of our study (OR = 1.70) because of differences criteria and disease diagnosis, but the difference in ORS between sexes is similar. A meta-analysis of depression and AD risk using random-effects models revealed pooled ORs of 2.03 for case-control and 1.90 for cohort studies.^26^ The OR for AD obtained in this study is also lower than that obtained in our study for dementia (OR = 2.637) because of differences in diagnosis. The pooled adjusted risk ratio (RR) for dementia when patients with DM were compared with those without dementia was 1.47.^28^ Another study based on the Taiwanese population has showed DM (hazard ratio [HR] = 1.76) to be associated with an increased risk for AD.^29^ A systematic literature review demonstrated that a history of stroke doubles the risk of incident dementia in the older population.^32^ In brief, the ORs of the 4 recognized risk factors for dementia obtained in our study are higher than those in other studies because we evaluated dementia whereas other studies evaluated AD.

We hypothesized that poor hearing (hearing loss) and poor eyesight (senile cataract) are associated with an increased risk of dementia in older people. A prospective population-based study in the United States demonstrated hearing loss to be an independent predictor for developing dementia (HR = 1.27, confidence interval [CI] = 1.03–1.56).^33^ Another preliminary investigation in Japan showed hearing loss to be independently associated with behavioral and psychological symptoms of dementia (OR = 4.65, 95% CI = 1.70–12.00).^34^ The OR of our study (OR = 1.557) is lower than that of the latter but higher than that of the former studies because these 3 studies evaluated different populations and had different statistical values (OR vs HR) and dementia diagnosis. The association between cataract and dementia has been controversial. According to animal model studies and pathological reviews, cataract is a potential biomarker for dementia because both these diseases share common etiological mechanisms.^35–37^ Moreover, a cataract surgery is associated with improving cognitive performance and decreasing dementia risk; it might cause the surgery to decrease the risk of fall injury and head injury.^38–40^ However, 2 other epidemiological studies have demonstrated a decreased association between cataract and dementia.^41,42^ Our study found that cataract is a potential risk factor for dementia in the Taiwanese population (OR = 1.492). One possible reason is that the severity of cataract and study population play major roles in the development of dementia. In brief, our results indicated that hearing loss and senile cataract are potential risk factors for dementia in the Taiwanese population.

Most research work is still based on classical frequentist hypothesis test. Hypothesis testing assesses the effect of a given risk factor by measuring OR and 95% CI to estimate the precision of OR. A large CI indicates a low precision level of OR, whereas a small CI indicates a high precision level of OR. The 95% CI does not report a measure's statistical significance. It would be inappropriate to interpret an OR with a 95% CI that spans the null value (eg, OR = 1), indicating lack of association between the risk factor and dementia. Because the frequentist interpretation of probability considers long-run frequencies rather than data at hand, it is impossible to assign a probability to a hypothesis, given the data. Frequentist hypothesis tests do not technically test any hypothesis, given the data; instead, the test the data, given the hypothesis. The Bayesians approach tests the hypothesis (ie, θ here), given the data.

Sample size that is too large or too small is a potential issue in classical frequentist hypothesis test. In the case of a too large sample size, hypothesis testing becomes biased in favor of rejecting the null hypothesis. When a sample size is too small, the frequentist reliance on asymptotic approximations becomes inappropriate and the estimates become biased. By contrast, Bayesian inference does not force an artificial dichotomy between a null and alternative hypothesis, allows exact probability statements about any hypothesis (ie, θ here), and is not biased due to a large sample size (eg, 1 million sample).^18^ Regarding a small sample size, Bayesian inference is also unbiased due to exact estimation (as opposed to asymptotic approximation) and still permits exact probability statements. Bayesian inference handles uncertainty better than frequentist inference, and the probability intervals of parameters are appropriately wide. In addition, the findings from the experimental result shown in Table 2 suggest that Bayesian inference can provide a similar trend as frequentist inference and thus can be helpful in assessing risks for dementia. Furthermore, because all observed data are used to compute posterior probability distribution, any bias from the data sampling, which is often criticized in frequentist hypothesis test, can be avoided. Furthermore, Bayesian approach is helpful in assessing the effect of the coexistence of multiple risk factors, which has not been discussed yet in research studies by using the analysis method of classical frequentist hypothesis test.

### Strengths and Limitations

The main strength of our study is the use of nationwide population-based data that accurately represent the general population. Majority of the events could be traced and referral bias is minimized because the Taiwan's NHI program is a single-payer, mandatory health insurance with affordable payments and services covering approximately 99% of the Taiwanese population. In addition, one of the major benefits of the Bayesian method is its ability to incorporate prior information.^43,44^ While other risk factor assessment approaches use prior information by illustrating the levels or ranges of individual parameters in sensitivity analysis, the Bayesian method analyzed historical data sets or refers expert and domain knowledge to determine what is known about biological parameters and processes.^18,45^ Most traditional risk factor assessment methods do not use any of the quantitative information that could be gathered from historical experiences with other risk factors and thus treat each risk factor assessment as a new and independent problem. However, it is extremely computationally intensive to apply Bayes theorem to complex models.^18,45^ It often takes days to conduct defensible decision analyses for assessments using computers. We tested consistency between results obtained using classical frequentist probability and Bayesian statistics in the 4 recognized risk factors for dementia (head injury, depression, DM, and vascular disease). Moreover, Bayesian statistics could effectively help clinicians and researchers to explore more potential risk factors for diseases like dementia using big data such as the Taiwan's NHIRD. Moreover, our results may provide a good representation of risk factors for patients with dementia in ethnic Chinese populations. Furthermore, the diagnoses of dementia and its comorbidities were reliable because these health insurance claims are scrutinized by medical reimbursement specialists and subject to peer review.

Nevertheless, there are some limitations of our study. First, the Taiwan's NHIRD does not include detailed information on biochemistry data, body mass index, clinical severity of diseases, family history, and lifestyle, which may be potential risk factors or comorbidities for dementia. As an example, *APOE* gene is recognized as a substantial factor in the majority of patients with dementia, but this biochemistry data are unavailable in the NHIRD. Similarly, lifestyle-related dementia risk factors, including physical activity, dietary habits, cigarette smoking, and alcohol consumption, were not available. Second, this study employed a retrospective study design, which tends to be more susceptible to biases than a prospective design^38^; therefore, we avoided 2 possible major biases related to sample problem (selecting random patients from nationwide data) and recall problem (analyzing national health care records). Finally, all administrative databases are subject to possible coding errors or undercoding.

## CONCLUSION

The findings of this study concur with our 2 hypotheses. First, our results of the 4 recognized risk factors for dementia, namely severe head injury, depression, DM, and vascular diseases, are consistent with both classical frequentist probability and Bayesian statistics. Second, hearing loss and senile cataract were found to be potential risk factors for dementia in the older Taiwanese population. Bayesian statistics could help clinicians to explore more risk factors for illnesses, such as dementia, to develop appropriate treatment strategies for these patients.

## Supplementary Material

Supplemental Digital Content

## References

[1] World Health Organization (WHO), 2012, http://www.who.int/mental_health/ental_health/publications/dementia_report_2012/en/ (online accessed on 25-Jun-2013).

[2] Klich-Rączka A Piotrowicz K Mossakowska M The assessment of cognitive impairment suspected dementia in Polish elderly people: results of the population-based PolSenior Study. *Exp Gerontol* 2014; 57:233–242. 24937034 10.1016/j.exger.2014.06.003

[3] Ferri CP Prince M Brayne C Global prevalence of dementia: a Delphi consensus study. *Lancet* 2005; 366:2112–2117. 16360788 10.1016/S0140-6736(05)67889-0 PMC2850264

[4] Hurd MD Martorell P Delavande A Monetary costs of dementia in the United States. *N Engl J Med* 2013; 368:1326–1334. 23550670 10.1056/NEJMsa1204629 PMC3959992

[5] Chen JH Lin KP Chen YC Risk factors for dementia. *J Formos Med Assoc* 2009; 108:754–764. 19864195 10.1016/S0929-6646(09)60402-2

[6] Ritchie K Carrie‘re I Ritchie CW Designing prevention programmes to reduce incidence of dementia: prospective cohort study of modifiable risk factors. *BMJ* 2010; 341:c3885. 20688841 10.1136/bmj.c3885 PMC2917002

[7] Pope SK Shue VM Beck C Will a healthy lifestyle help prevent Alzheimer's disease? *Annu Rev Public Health* 2003; 24:111–132. 12415146 10.1146/annurev.publhealth.24.100901.141015

[8] Guidance for the use of Bayesian statistics in medical device clinical trials. Guidance for industry and FDA staff, 2010, http://www.fda.gov/downloads/MedicalDevices/DeviceRegulationandGuidance/GuidanceDocuments/ucm071121.pdf (cited in 2016).

[9] Ashby D Bayesian statistics in medicine: a 25 year review. *Stat Med* 2006; 25:3631–3589. 10.1002/sim.2672 16947924

[10] Neugroschl JA Kolevzon A Samuels SC Sadock BJ Sadock VA Dementia. *Kaplan & Sadock's Comprehensive Textbook of Psychiatry, 8th ed., Vol. 1.*. Baltimore: Lippincott Williams & Wilkins; 2005 1068.

[11] Cheng TM Okma KGH Crivelli L Taiwan's National Health Insurance System: High Value for the Dollar. *Six Countries, Six Reform Models: The Health Reform Experience of Israel, the Netherlands, New Zealand, Singapore, Switzerland and Taiwan.*. New Jersey: World Scientific; 2009 71–204.

[12] Wong CS Lin YC Hong LY Increased long-term risk of dementia in patients with carbon monoxide poisoning: a population-based study. *Medicine (Baltimore)* 2016; 95:e2549. 26817904 10.1097/MD.0000000000002549 PMC4998278

[13] Vinogradova Y Hippisley-Cox J Coupland C Identification of new risk factors for pneumonia: population-based case-control study. *Br J Gen Pract* 2012; 59:329–338. 10.3399/bjgp09X472629 PMC2751937 19843413

[14] Lee YC Lin CH Wu RM Discontinuation of statin therapy associates with Parkinson disease: a population-based study. *Neurology* 2013; 81:410–416. 23884037 10.1212/WNL.0b013e31829d873c

[15] Tsai CF Liu CJ Chen TJ Increased incidence of orthopedic fractures in cirrhosis patients: a nationwide population-base study. *J Hepatol* 2013; 58:706–714. 23238105 10.1016/j.jhep.2012.12.001

[16] Torres A Peetermans WE Viegi G Risk factors for community-acquired pneumonia in adults in Europe: a literature review. *Thorax* 2013; 68:1057–1065. 24130229 10.1136/thoraxjnl-2013-204282 PMC3812874

[17] DeGroot MH Schervish MJ Probability and Statistics. 4th ed 2011; New York: Pearson, ISBN-13: 978-0321500465.

[18] Howson C Urbach P Bayesian reasoning in science. *Nature* 1991; 350:371–374.

[19] Kalaria RN Maestre GE Arizaga R Alzheimer's disease and vascular dementia in developing countries: prevalence, management, and risk factors. *Lancet Neurol* 2008; 7:812–826. 18667359 10.1016/S1474-4422(08)70169-8 PMC2860610

[20] Berr C Wancata J Ritchie K Prevalence of dementia in the elderly in Europe. *Eur Neuropsychopharmacol* 2005; 15:463–471. 15955676 10.1016/j.euroneuro.2005.04.003

[21] Lobo A Launer LJ Fratiglioni L Prevalence of dementia and major subtypes in Europe: a collaborative study of population-based cohorts. Neurologic Diseases in the Elderly Research Group. *Neurology* 2000; 54:S4–S9. 10854354

[22] Hofman A Rocca WA Brayne C The prevalence of dementia in Europe: a collaborative study of 1980–1990 findings. Eurodem Prevalence Research Group. *Int J Epidemiol* 1991; 20:736–748. 1955260 10.1093/ije/20.3.736

[23] Fleminger S Oliver DL Lovestone S Head injury as a risk factor for Alzheimer's disease: the evidence 10 years on; a partial replication. *J Neurol Neurosurg Psychiatry* 2003; 74:857–862. 12810767 10.1136/jnnp.74.7.857 PMC1738550

[24] Reitz C Mayeux R Alzheimer disease: epidemiology, diagnostic criteria, risk factors and biomarker. *Biochem Pharmacol* 2014; 88:640–651. 24398425 10.1016/j.bcp.2013.12.024 PMC3992261

[25] Lee YK Hou SW Lee CC Increased risk of dementia in patients with mild traumatic brain injury: a nationwide cohort study. *PLoS One* 2013; 8:e62422. 23658727 10.1371/journal.pone.0062422 PMC3641064

[26] Ownby RL Crocco E Acevedo A Depression and risk for Alzheimer disease: systematic review, meta-analysis, and metaregression analysis. *Arch Gen Psychiatry* 2006; 63:530–538. 16651510 10.1001/archpsyc.63.5.530 PMC3530614

[27] Chen MH Li CT Tsai CF Risk of subsequent dementia among patients with bipolar disorder or major depression: a nationwide longitudinal study in Taiwan. *J Am Med Dir Assoc* 2015; 16:504–508. 25737262 10.1016/j.jamda.2015.01.084

[28] Lu FP Lin KP Kuo HK Diabetes and the risk of multi-system aging phenotypes: a systematic review and meta-analysis. *PLoS One* 2009; 4:e4144. 19127292 10.1371/journal.pone.0004144 PMC2607544

[29] Huang CC Chung CM Leu HB Diabetes mellitus and the risk of Alzheimer's disease: a nationwide population-based study. *PLoS One* 2014; 9:e87095. 24489845 10.1371/journal.pone.0087095 PMC3906115

[30] Chao TF Liu CJ Chen SJ Statins and the risk of dementia in patients with atrial fibrillation: a nationwide population-based cohort study. *Int J Cardiol* 2015; 196:91–97. 26080283 10.1016/j.ijcard.2015.05.159

[31] Hsu CY Huang CC Chan WL Angiotensin-receptor blockers and risk of Alzheimer's disease in hypertension population—a nationwide cohort study. *Circ J* 2013; 77:405–410. 23149416 10.1253/circj.cj-12-0658

[32] Sawa GM Stephan BC Alzheimer's Society Vascular Dementia Systematic Review Group. Epidemiological studies of the effect of stroke on incident dementia: a systematic review. *Stroke* 2010; 41:e41–e46. 19910553 10.1161/STROKEAHA.109.559880

[33] Gurgel RK Ward PW Schwartz S Relationship of hearing loss and dementia: a prospective, population-based study. *Otol Neurotol* 2014; 35:775–781. 24662628 10.1097/MAO.0000000000000313 PMC4024067

[34] Umeda-Kameyama Y Iijima K Yamaguchi K Association of hearing loss with behavioral and psychological symptoms in patients with dementia. *Geriatr Gerontol Int* 2014; 14:727–728. 25065402 10.1111/ggi.12185

[35] Goldstein LE Muffat JA Cherny RA Cytosolic beta-amyloid deposition and supranuclear cataracts in lenses from people with Alzheimer's disease. *Lancet* 2003; 361:1258–1265. 12699953 10.1016/S0140-6736(03)12981-9

[36] Harding JJ Cataract, Alzheimer's disease, and other conformational diseases. *Curr Opin Opthalmol* 1998; 9:10–13. 10.1097/00055735-199802000-00003 10178625

[37] Melov S Wolf N Strozyk D Mice transgenic for Alzheimer disease beta-amyloid develop lens cataracts that are rescued by antioxidant treatment. *Free Radic Biol Med* 2005; 38:258–261. 15607908 10.1016/j.freeradbiomed.2004.10.023

[38] Yu WK Chen YT Wang SJ Cataract surgery is associated with a reduced risk of dementia: a nationwide population-based cohort study. *Eur J Neurol* 2015; 22:1370–1380. 25196252 10.1111/ene.12561

[39] Jefferis JM Taylor JP Collerton J The association between diagnosed glaucoma, cataract and cognitive performance in very old people: cross-sectional finding from the Newcastle 85+ study. *Opthalmic Epidemiol* 2013; 20:82–88. 10.3109/09286586.2012.757626 PMC4121672 23510311

[40] Tseng VL Yu F Lum F Risk of fracture following cataract surgery in medicare beneficiaries. *JAMA* 2012; 308:493–501. 22851116 10.1001/jama.2012.9014

[41] McGwin G Hall TA Searcey K Cataract and cognitive function in older adults. *J Am Geriatr Soc* 2005; 53:1260–1261. 16108952 10.1111/j.1532-5415.2005.53384_2.x

[42] Grodstein F Chen J Hankinson SE Cataract extraction and cognitive function in older women. *Epidemiology* 2003; 14:493–497. 12843777 10.1097/01.ede.0000083503.34133.8c

[43] Niragire F Achia TN Lyambabaje A Bayesian mapping of HIV infection among women of reproductive age in Rwanda. *PLoS One* 2015; 10:e0119944. 25811462 10.1371/journal.pone.0119944 PMC4374935

[44] Wang XH Zheng B Good WF Computer-assisted diagnosis of bread cancer using a data-driven Bayesian belief network. *Int J Med Inform* 1999; 54:115–126. 10219951 10.1016/s1386-5056(98)00174-9

[45] Darwiche A Bayesian networks. *Commun ACM* 2010; 53:80–90.

