# Supplementary material for: A Bayesian Approach to Identifying New Risk Factors for Dementia: A Nationwide Population-Based Study
Source: Medicine (Baltimore). 2016 May 27;95(21):e3658. doi: 10.1097/MD.0000000000003658 (PMC4902349; doi:10.1097/MD.0000000000003658)
Supplement: Supplemental Digital Content [file medi-95-e3658-s001.doc]

**TABLE e-1. ICD-9-CM codes and anatomical therapeutic chemical [ATC] classification system codes used in this study**

| **Main Diseases** | **ICD-9-CM codes and A-codes** |
| --- | --- |
| Dementia | 290, 294.1-294.2, 331.0, A210, A213, A222 |
| **Comorbidities** | **ICD-9-CM codes and A-codes** |
| Severe head injury | 431, 430, 800-804, 850-852, 853.1-853.2, 854.0-854.1, A290, A291, A470 |
| Depression | 296.2-296.3, 296.82, 293.83, 300.4, 311 |
| Diabetes mellitus | 249-250, 648.01, 648.02, 588.1, 357.2, A181 |
| Vascular diseases | 325, 430-459, 410, 411.0, 411.1, 411.81, 411.89, 412, 413.1, 413.9, 414.00-414.07, 414.10-414.12, 414.19, 414.2-414.4, 414.8, 414.9, A279, A291-A294, A299 |
| Senile cataract | 366, A231 |
| Hearing loss | 389, A241 |
